# B7‐1 and programmed cell death‐ligand 1 in primary and lymph node metastasis lesions of non‐small cell lung carcinoma

**DOI:** 10.1002/cam4.4444

**Published:** 2021-12-14

**Authors:** Takehiro Yamada, Yasuhiro Miki, Miho Suzuki, Osamu Kondoh, Ryoko Saito‐Koyama, Katsuhiko Ono, Yoshinori Okada, Hironobu Sasano

**Affiliations:** ^1^ Department of Thoracic Surgery Institute of Development, Aging and Cancer Tohoku University Sendai Japan; ^2^ Department of Pathology Tohoku University Graduate School of Medicine Sendai Japan; ^3^ Product Research Department Chugai Pharmaceutical Co., Ltd Kamakura Japan

**Keywords:** B7‐1, immune check point, lymph node metastasis, non‐small cell lung cancer, PD‐L1

## Abstract

**Background:**

Programmed cell death ligand 1 (PD‐L1) status has been reported to be different between metastatic and primary lesions in some cases. Therefore, the interaction between carcinoma and immune cells could influence their expression in the tumor microenvironment. PD‐L1 is known to bind not only to Programmed cell death 1 (PD‐1) but also to B7‐1 (CD80). In this study, we examined the interaction between lung carcinoma cell lines and peripheral blood mononuclear cells (PBMCs) in vitro. We then examined the significance of B7‐1 expression non‐small cell lung cancer (NSCLC) microenvironment.

**Methods:**

The interaction of lung carcinoma cell lines and PBMC through the soluble factors was analyzed using a co‐culture system. The changes in expression of immune checkpoint‐related factors in PBMC were examined by PD‐1/PD‐L1 Checkpoint Pathway qPCR Array Kit. B7‐1 expression in NSCLC tissues was examined by immunohistochemistry.

**Results:**

B7‐1 was upregulated following the co‐culture with the lung carcinoma cell lines. B7‐1 expression in NSCLC tissues was significantly higher in smokers and squamous cell carcinomas and was significantly positively correlated with PD‐L1 status in primary cancer. However, B7‐1 and PD‐1 were not correlated between primary and metastatic diseases in the same patients.

**Conclusion:**

PD‐1 inhibitors inhibited PD‐1/PD‐L1 binding but not PD‐L1/B7‐1 binding. These results demonstrated that the intratumoral ratio of B7‐1 positive T cells in NSCLC tissue could be involved in the therapeutic efficacy of PD‐L1 inhibitors. This study focused on lymph node metastasis but other sites of distant metastases should be explored by further analysis.

## Graphical abstract

Fluorescence microscopic representative illustration of CD4, CD8, and B7‐1 immunohistochemistry.

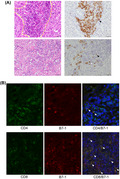

## 1 INTRODUCTION

Immune checkpoint inhibitors have received enormous attention as one of novel therapeutic approaches of cancer patients. In particular, some programmed cell death 1 (PD‐1)/programmed cell death‐ligand 1 (PD‐L1) inhibitors have been clinically available for medical therapy of non‐small cell lung cancer (NSCLC) patients.^1^, ^2^ PD‐L1, also known as B7‐H1 and CD274, is a membrane protein normally expressed primarily in antigen‐presenting cells and suppresses T‐cell activation by binding to PD‐1,^3^ also known as a CD279, present in T cells.^3^ Some carcinoma cells have been well known to express PD‐L1 in order to avoid immune‐mediated attacks from immune system against them.^4^ PD‐1/PD‐L1 inhibitors are also well known to restore cancer immunity by inhibiting the direct binding of PD‐1 and PD‐L1 ^5^ and to be more effective against many PD‐L1‐bearing cancers.^5^ However, it is also true that PD‐L1 status was reported to be sometimes altered from primary to metastatic lesions of the very same lung cancer patients.^6^, ^7^ This discrepancy of PD‐L1 expression between primary and metastatic lesions of NSCLC has been considered to represent the spectrum of intra‐tumoral heterogeneity.^8^ In addition, the possible effects of the soluble factors secreted from immune cells could influence the PD‐L1 expression in carcinoma cells located in the lymph node metastatic by these soluble factors above. PD‐L1 is well known to be induced by some cytokines secreted from tumor‐infiltrating lymphocytes (TILs),^9^ and the presence of abundant PD‐1‐positive T cells was also reported to be located around PD‐L1‐positive carcinoma cells.^10^ These results all suggest that the changes in PD‐L1 status detected in metastases could be related to TILs and their PD‐1 status.

PD‐L1 is known to bind not only to PD‐1 but also to B7‐1 (CD80), which is located in the cell membrane of immune cells such as dendritic and T cells.^11^ B7‐1 binds to CD28 in T cells to subsequently activate T cells and suppresses T cells by binding to CTLA‐4.^12^ B7‐1 on T cells is known to convey inhibitory signals to T cells by binding to PD‐L1 as in PD‐1.^11^ Therefore, B7‐1 status is reasonably postulated to be correlated with PD‐L1 as in the case of PD‐1. In addition, the intratumoral status of B7‐1 could also influence the therapeutic effects of PD‐1 inhibitor in NSCLC patients. PD‐1 inhibitor is also reasonably postulated to inhibit PD‐1/PD‐L1 but by no means B7‐1/PD‐L1 binding, because it binds to only PD‐1 but not PD‐L1. Therefore, the intratumoral presence or abundance of B7‐1 is also considered to influence the therapeutic efficacy of PD‐1 but not PD‐L1 inhibitors, which could contribute to the selection of immune check point inhibitors. However, the status of B7‐1 and its clinical significance in NSCLC tissues have remained virtually unknown.

The assessment of PD‐L1 status in carcinoma cells was usually performed only in primary or metastatic NSCLC cell to determine the possible therapeutic efficacy of PD‐1/PD‐L1 inhibitors.^13^ However, it is also important to evaluate its status in both primary and metastatic lesions or at least to have the information as to the degrees of potential differences between primary and metastatic NSCLC lesions in order to achieve the maximum therapeutic efficacy of the patients. Therefore, in this study, we first explored the possible interaction between lung carcinoma cells and lymphocytes through the soluble factors using in vitro co‐culture system. We examined the changes in expression status of immune check point‐related molecules in peripheral blood mononuclear cells co‐cultivated with lung carcinoma cell lines using PCR array. We then immunolocalized B7‐1 and PD‐L1 in both primary to metastatic NSCLC lesions of the patients and compared the findings between them with correlation to the status of immune cells around carcinoma cells to further explore the clinicopathological significance of B7‐1 in NSCLC.

## 2 MATERIALS AND METHODS

### 2.1 Carcinoma cell lines

Human lung adenocarcinoma cell lines, PC‐9, A549, and H1975 were all purchased from American type culture collection (Virginia). Human peripheral blood mononuclear cells (PBMCs) derived from a 50‐year‐old healthy male donor were obtained from Precision Bioservices. All these cells above were cultured in RPMI1640 (Sigma‐Aldrich) at 37℃ in humidified incubator containing 5% CO_2_.

The interaction of lung carcinoma cells and PBMC above through the soluble factors was analyzed using a co‐culture system.^14^ In this study, we used 6‐Well ThinCert (membrane pore size, 0.4 µm) (Greiner Bio‐One, Kremsmünster) for co‐culture. Lung carcinoma cells and PBMC were both cultured in inserts and wells of ThinCert, respectively, and RNA was extracted from PBMC with TRI reagent (Cosmo Bio) after 24 h of co‐culture.

### 2.2 PCR array

Total RNA was reverse transcribed into cDNA using QuantiTect Reverse Transcriptional Kit (Qiagen, Hilden). In this study, GeneQuery Human PD‐1/PD‐L1 Checkpoint Pathway qPCR Array Kit (ScienCell Research Laboratories) was used to study the changes in the expression of immune checkpoint‐related factors in PBMC. Quantitative PCR was performed according to the qPCR Array Kit manual (https://www.sciencellonline.com/PS/GK121.pdf) using LightCycler 96 (Roche Diagnostics). The control is cDNA from the PBMCs cultured alone for 24 h. Table 1 summarizes the gene lists measured by the PCR array. The ratio of change in the target gene to the control was calculated according to the kit manual (https://www.sciencellonline.com/PS/GK121.pdf) as follows: The difference between the values of each gene after co‐culture and the control was calculated and defined as ΔCq. The average of ΔCq of the five housekeeping genes (*ACTB*, *GAPDH*, *LDHA*, *NONO*, and *PPIH*) was calculated and defined as ΔCq(ref). The difference between the expression of the target gene [ΔCq(Target)] and ΔCq(ref) was determined and defined as ΔΔCq(Target). The corrected amount of change in the target gene is 2^−ΔΔCq(Target)^ (fold change).

**TABLE 1:** Summary of the genes evaluated by the PCR array

Co‐stimulatory/inhibitory receptor	*PDCD1*(PD‐1), *CD28*, *CD27*, *CD276*
	*ICOS*, *TNFRSF18*, *TNFRSF4*, and *TNFRSF9*
Co‐stimulatory/inhibitory pathway	*CD274*(PD‐L1), *PDCD1LG2*(PD‐L2)
	*CD4*, *LCK*, *CD2*, *CD247*, *CSK*
	*PTPN11*, *PTPN6*, and *NCOR2*
PD‐L1 induction	*IFNy*, *IL15*, *IL17A*, *IL2*, *IL21*, *IL7*
	*MAPK1*, *NRP1*, and *STAT3*
T‐cell receptor signaling	*AKT1*, *CD3*, *CTLA4*, *CD8*, *GSK3B*
	*JUN*, *NFKB1*, and *PDPK1*
T‐cell function regulation	*BATF*, *FOXO1*, *KRAS*, *MAPK8*, *MTOR*
	*NFATC1*, *PI3Ks*, *SKP2*, and *TLR9*
B‐cell function regulation	*CD80*(B7‐1), *CD86*, and *POU2F2*
Pro‐inflammatory molecules	*CSF2*, *IL4*, *IL10*, *IRF6*, *TNF*, and *VEGFA*
Additional immune checkpoint genes	*HAVCR2*, *HNF1A*, *LAG3*, and *TIGHT*
Housekeeping genes	*ACTB*, *GAPDH*, *LDHA*, *NONO*, and *PPIH*

Abbreviations: ACTB, beta‐actin; BATF, basic leucine zipper ATF‐like transcription factor; CD, cluster of differentiation; CSF2, colony‐stimulating factor 2; CSK, C‐terminal Src kinase; CTLA4, cytotoxic T‐lymphocyte antigen 4; FOXO1, forkhead box protein O1; GAPDH, glyceraldehyde 3‐phosphate dehydrogenase; GSK3B, glycogen synthase kinase 3 beta; HAVCR2, hepatitis A virus cellular receptor 2; HNF1A, hepatocyte nuclear factor 1‐alpha; ICOS, inducible T‐cell co‐stimulatory; IL, interleukin; INFG, Interferon gamma; IRF6, interferon regulatory factor 6; JUN, c‐Jun; KRAS, K‐ras; LAG3, lymphocyte activation gene 3; LCK, lymphocyte cell‐specific protein‐tyrosine kinase; LDHA, lactate dehydrogenase A; MAPK, mitogen‐activated protein kinase; MTOR, mammalian target of rapamycin; NCOR, nuclear receptor corepressor; NFATC1, nuclear factor of activated T cells 1; NFKB1, nuclear factor kappa B subunit 1; NONO, non‐POU domain containing, octamer‐binding; NRP1, neuropilin 1; PDCD1, programmed cell death 1; PDCD1LG2, programmed cell death 1 ligand 2; PDPK1, phosphoinositide‐dependent kinase 1; PI3Ks, phosphoinositide 3‐kinases; POU2F2, POU class 2 homeobox 2; PPIH, peptidyl isomerase H; PTPN, protein tyrosine phosphatase non‐receptor type; SKP2, S‐phase kinase‐associated protein 2; STAT3, signal transducer and activator of transcription 3; TIGHT, T‐cell immune receptor with Ig and ITIM domains; TLR9, toll‐like receptor 9; TNF, tumor necrosis factor; TNSF, tumor necrosis factor receptor superfamily; VEGFA, vascular endothelial growth factor A.

### 2.3 NSCLC cases

Surgical pathology specimens of NSCLC patients who underwent lung resection at Tohoku University Hospital were retrieved from pathology files of Tohoku University Hospital. Thirty‐five cases were randomly selected (tentatively termed Group A), and 40 from the patients who underwent both lung resection and lymph node dissection at the time of surgery, and foci of carcinoma metastases with tumor stroma were histologically confirmed in the dissected lymph nodes away from primary tumor (tentatively termed Group B). A total of 75 cases on NSCLC were examined in this study. Tables 2 and 3 summarize the clinicopathological finding of all the cases examined and Group B, respectively. In lung cancer treatment in Japan, 70 years of age had been used as the cutoff value for elderly patients, but this age was revised to 75 years in 2012.^15^ As most of the cases examined in this study were before 2012, the cutoff value was tentatively set as 70 years in this study. All the specimens had been fixed in 10% neutral buffered formalin aqueous solution and paraffin‐embedded.

**TABLE 2:** The clinicopathological factors and B7‐1 positive rate in 75 cases

	Number of cases (%)	B7‐1 positive rate (%) (Mean ± SD)	*p* value
Age			
Mean ± SD	65.93 ± 9.32	18.49 ± 14.57	
70 y.o. >	47 (62.7)	20.00 ± 16.36	0.443
70 y.o. ≦	28 (37.3)	15.96 ± 10.73	
Sex			
Male	47 (62.7)	19.76 ± 16.41	0.576
Female	28 (37.3)	16.37 ± 10.73	
Smoking^a^			
Non‐smoker	26 (22.9)	12.74 ± 7.93	**0.018**
(Ex‐) smoker	49 (77.1)	21.55 ± 16.34	
Histologic type			
Adenocarcinoma	54 (72.0)	15.18 ± 10.61	**0.008**
Squamous cell carcinoma	21 (28.0)	27.01 ± 19.51	
Pathological stage			
Ⅰ	22 (29.7)	16.70 ± 14.63	0.428
Ⅱ+Ⅲ+Ⅳ	52 (70.3)	18.96 ± 14.62	
Ⅱ	17 (23.0)		
Ⅲ	30 (40.5)		
Ⅳ	5 (6.8)		
Unknown	1		
EGFR mutation (adenocarcinoma only)			
Positive	9 (16.6)	12.67 ± 3.58	
Negative	17 (31.5)	13.94 ± 10.78	0.788
Unknown	28 (51.9)		

Abbreviations:

SD, standard deviation.

^a^ Brinkman index = Number of smokers per day × Years of smoking >300: (Ex‐) smoker.

Bold is *p* <0.05.

**TABLE 3:** The clinicopathological factors of Group B patients (40 cases who have lymph node metastases).

	Number of cases (%)
Age	
Mean ± SD	67.08 ± 8.86
70 y.o. >	23 (57.5)
70 y.o. ≦	17 (42.5)
Sex	
Male	20 (50.0)
Female	20 (50.0)
Smoking	
Non‐smoker	17 (42.5)
(Ex‐) smoker	23 (57.5)
Histologic type	
Adenocarcinoma	30 (75.0)
Squamous cell carcinoma	10 (25.0)
Pathological stage	
Ⅱ	10 (25.0)
Ⅲ	26 (65.0)
Ⅳ	4 (10.0)
EGFR mutation (adenocarcinoma only)	
Positive	9 (30.0)
Negative	17 (56.7)
Unknown	4 (14.3)

### 2.4 Immunohistochemistry

In group A, immunohistochemistry of B7‐1 (CD80) alone was performed. In Group B (40 cases with lymph node metastases), B7‐1, PD‐1, CD3, CD4, CD8, and PD‐L1 were all immunolocalized in primary lesions and lymph node metastases. Table 4 summarizes the details of primary antibodies used for immunohistochemistry.^14^, ^16^ Immunohistochemistry was performed as follows: First, the specimens were autoclaved at 120°C for 5 min for antigen retrieval. Streptavidin–biotin amplification method with Histofine Kit (Nichirei)^14^ was employed as immunostaining. Colorimetric reaction was performed using 3,3‐diaminobenzidine (DAB) and counterstained with hematoxylin. Immunohistochemistry of PD‐L1 was performed with Ventana OptiView PD‐L1: SP263 and BenchMark Ultra (both Roche Diagnostics).^14^

**TABLE 4:** Monoclonal antibodies used for immunohistochemistry

Antigen	Origin creature	Final dilution ratio	Positive control	Manufacturer	Clone no.
CD80 (B7‐1)	Mouse	x1000	Tonsil	R&D Systems	37711
PD‐1	Mouse	x100	Tonsil	Abcam	NAT105
CD3	Mouse	x500	Lymph node	DAKO	F7.2.38
CD4	Rabbit	x400	Lymph node	Abcam	EPR6855
CD8	Mouse	x50	Lymph node	DAKO	C8/144B
PD‐L1	Rabbit	—	—	Roche	SP263

Note.

R&D Systems, Minneapolis, USA; Abcam, Cambridge, USA; DAKO, Carpentaria, USA; Roche, Roche Diagnostics, Mannheim, Germany.

The determination of histological features of immunopositive cells was visually performed by comparing serial hematoxylin‐eosin (HE) stained slides with care. The number of B7‐1, PD‐1, CD3, CD4, or CD8‐positive cells was evaluated using the automatic analysis software HALO (Indica labs)^17^ after capturing the images through virtual microscopy as follows: Approximately 1 mm^2^ region of intratumoral stromal area was randomly selected after glancing the whole specimen. The positive cells in the region were subsequently discriminated with automatic analysis. “Positive cells/total number of cells in the selected tumor stroma” was defined as “positive rate.” The ratio of PD‐L1‐positive cells to all carcinoma or tumor cells (Tumor proportion score: TPS) was then obtained.^18^ Regardless of immune intensity, the cells with membranous immunoreactivity were regarded as positive. PD‐L1 TPS was evaluated from 0% to 100%, with 10% increments.

In this study, we also immunolocalized PD‐L1 in lymph node without cancer metastasis of NSCLC patients. SP142 was used to immunolocalize PD‐L1 in immune cells because of its reported high specificity and sensitivity.^19^, ^20^ Therefore, in this study, SP142 antibody was used to study the localization of PD‐L1‐positive immune cells in non‐metastatic lung lymph nodes. Immunohistochemistry with Ventana OptiView PD‐L1: SP142 and BenchMark Ultra (both Roche Diagnostics) has been optimized to evaluate PD‐L1 staining on both carcinoma cells and immune cells.^19^

### 2.5 Immunofluorescence

Forty cases of primary NSCLC tissues were studied with immunofluorescence. In the immunofluorescence, double staining of CD4/B7‐1 and CD8/B7‐1 was examined to identify the B7‐1‐positive T‐cell subtype. The primary antibodies of CD4, CD8, and B7‐1 were as follows: CD4, rabbit monoclonal EPR6855 (Abcam); CD8; CD8, rabbit monoclonal SP‐16 (Abcam); and B7‐1, mouse monoclonal 37711 (R&D Systems, Minneapolis, USA). The two antibodies were simultaneously treated in the tissue section and incubated overnight at 4°C. The specimens were then reacted with corresponding secondary antibodies for 1 h at room temperature. The secondary antibodies employed in this study were anti‐mouse antibody Alexa Fluor 594 and anti‐rabbit antibody Alexa Fluor 488 (Thermo Fisher Scientific). The number of each lymphocyte marker and those co‐localized with B7‐1 was counted under a fluorescence microscope (BZ‐9000, Keyence).

### 2.6 Statistical analysis

Clinicopathological factors and the positive rate in immunohistochemistry were evaluated with the chi‐squared test and the Wilcoxon signed‐rank test. Multivariate analysis of the positive rate of each molecule was performed with multiple regression. Pathological stage and the age were both analyzed as reported,^21^ tentatively divided into two groups, Stage I and Stage II–IV, 70 years or younger and older than 70 years old. All the statistical analyses were performed using statistical analysis software JMP13.1.0 (SAS Institute) and a *p* value of <0.05 was evaluated as significant in this study.

## 3 RESULTS

### 3.1 PBMC genes increased after co‐culture with lung adenocarcinoma cells

**FIGURE 1:**
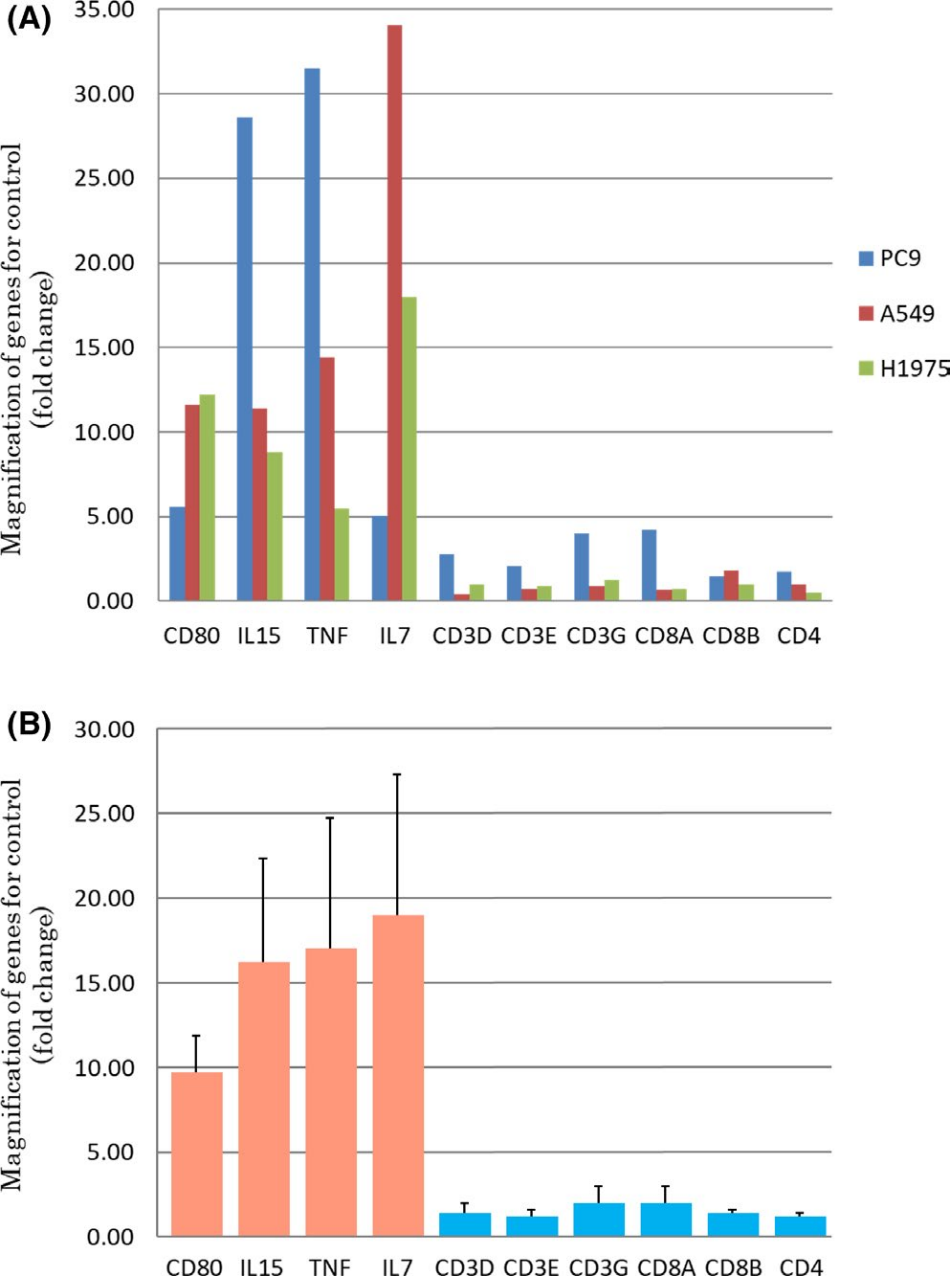
PBMC genes that increased by more than fivefold following the co‐culture with pulmonary adenocarcinoma cells. The ordinate represents the fold changes in gene expression of PBMCs following the co‐culture with lung adenocarcinomas cells (PC‐9, A549, and H1975) for 24 h. The fold change for each gene is summarized in a bar graph. A represents the value for each of the three cell lines and B represents the mean value (error bars, standard errors) of the three cell lines. We selected the upregulated genes that increased with more than fivefold in co‐culture with any of these three lung carcinoma cell lines, and the genes which increased with more than fivefold are CD80, IL‐15, TNF, and IL‐7

The status of immune check point‐related genes in PBMC after co‐cultured with lung carcinoma cells (PC‐9, A549, and H1975) for 24 h was compared with the control (PBMC cultured alone for 24 h. The genes which elevated with more than fivefold compared to the control after co‐culture were *CD80* (B7‐1), *IL*‐*7*, *IL*‐*15*, and *TNF*. Figure 1 illustrates the fold changes in individual genes above. Any of the genes encoding T‐cell markers, CD3, CD4, and CD8 including their isoforms also increased in less than fivefold compared to the control. No significant changes were detected in *PD*‐*1* expression of PBMC in any of the co‐culture conditions examined in this study.

### 3.2 The characteristics of B7‐1‐positive cells

**FIGURE 2:**
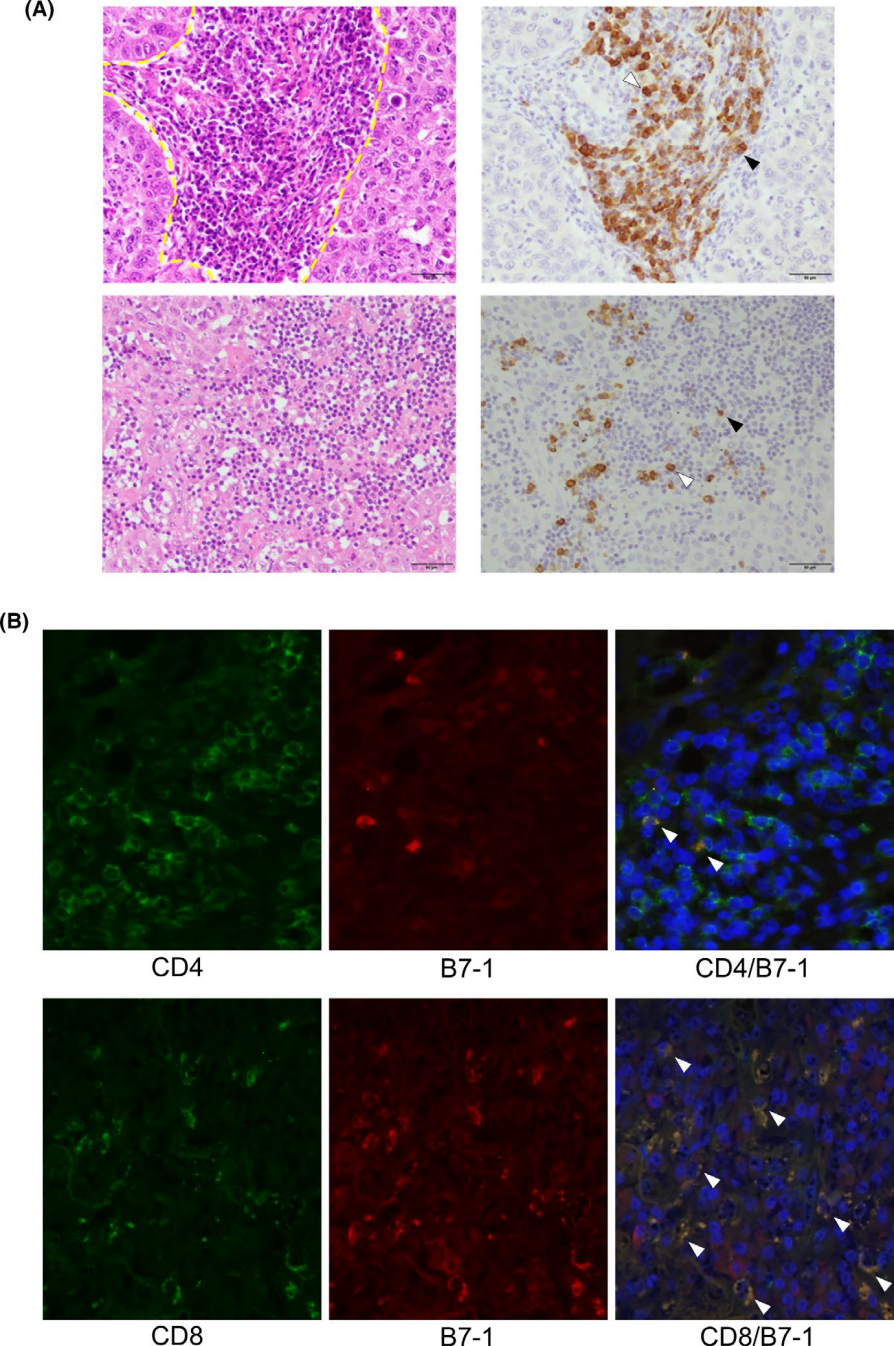
Identification of B7‐1‐positive cells in immunohistochemistry. (A) Light microscopic representative illustration of hematoxylin and eosin staining (*left*) and B7‐1 immunohistochemistry (*right*). The tumor stroma is inside the yellow dashed line in HE figure B7‐1 immunoreactivity is detected in the cytoplasm of tumor stromal cells. The careful comparison of the findings with serial tissue hematoxylin‐eosin stained sections revealed that these positive cells are either lymphocytes (black arrowheads) or plasma cells (white arrowheads). Primary site of lung adenocarcinoma (*upper*) and its lymph node metastatic site (*lower*) are demonstrated. (B) Fluorescence microscopic representative illustration of CD4 (*upper left*, *green*), CD8 (*lower left*, *green*), and B7‐1 immunohistochemistry (*upper and lower middle*, *red*). Merged image is also demonstrated (*upper and lower right*). Double positive cells (*yellow*) are indicated by arrowheads. DAPI is used as a nuclear counterstain (*upper and lower right*, *blue*)

Representative images of B7‐1 immunohistochemistry and HE staining of serial tissue sections are illustrated in Figure 2A. B7‐1 immunoreactivity was detected in the cytoplasm of the cells present in tumor stroma. Analysis of serial tissue sections revealed that those B7‐1‐positive cells were morphologically determined as lymphocytes or plasma cells. Representative images of double immunofluorescence of CD4/B7‐1 and CD8/B7‐1 are illustrated in Figure 2B. The proportion of B7‐1‐positive cells in CD8‐positive T cells was significantly higher than those in CD4‐positive T cells in immunofluorescence analysis (mean ± standard division %, CD4/B‐7‐1 6.23 ± 5.06; CD8/B7‐1 20.44 ± 10.04; *p* < 0.0001).

### 3.3 The correlation of B7‐1 with clinicopathological factors of NSCLC patients

The correlation between B7‐1 positive rate and clinicopathological factors in 75 cases of NSCLC patients is summarized in Table 2. The mean ± SD (standard deviation) of the B7‐1 positive rates was 18.49% ± 14.57%. The B7‐1 positive rate was significantly higher in smokers than in nonsmokers (*p *= 0.018) and in squamous cell carcinoma than in adenocarcinoma (*p *= 0.008). In this study, there were no significant correlations between EGFR mutations and B7‐1 expression in lung adenocarcinoma (*p *= 0.788). There were also no significant differences in B7‐1 (exon 19 deletion, 13.00 ± 4.36; L858R mutation, 12.03 ± 1.72; *p *= 0.728) and PD‐L1 (exon 19 deletion, 0.55 ± 0.26; L858R mutation, 0.22 ± 0.27; *p *= 0.427) expression between the two EGFR mutations (exon 19 deletion, *n* = 6; L858R mutation, *n* = 3). No other significant correlation was detected among pathological stage, gender, and age.

### 3.4 Association among the positive rates of each molecules examined

The correlation among smoking (Brinkman index), age, B7‐1 positive rate, PD‐1 positive rate, CD3 positive rate, CD4 positive rate, CD8 positive rate, and PD‐L1 TPS was evaluated using multivariate analysis with multiple regression in Group B (lung primary cancer, Table 5; lymph node metastasis, Table 6). In lung primary carcinomas, statistically significant positive correlation was detected between PD‐L1 TPS and B7‐1 positive rate, PD‐L1 TPS and PD‐1 positive cell rate, and PD‐L1 and smoking amount. A significant positive correlation was also detected between B7‐1 positive cell rate and Brinkman index of the patients examined. A significant negative correlation was detected between Brinkman index of the patients and CD3 or CD4 positive rate, respectively. In lymph node metastases, a significant positive correlation was detected between PD‐L1 TPS and Brinkman index, but no significant correlation was detected among PD‐L1 TPS, B7‐1, and PD‐1 positive rates.

**TABLE 5:** Correlation between immunohistochemical positive rate, Brinkman index, and age of the patients (Primary cancer)

	Age	Smoking amount	PD‐L1	B7‐1	PD‐1	CD3	CD8	CD4
Age		*r* = 0.116 *p *= 0.477	*r* = −0.055 *p *= 0.735	*r* = 0.066 *p* = 0.686	*r* = −0.042 *p* = 0.799	*r* = 0.071 *p* = 0.624	*r* = −0.008 *p* = 0.962	*r* = −0.055 *p* = 0.735
Smoking amount (Brinkman index)	*r* = 0.116 *p* = 0.477		***r*** = **0.672** ***p *< 0.001**	***r*** = **0**.**461** ***p*** = **0.003**	*r* = 0.104 *p* = 0.524	***r*** = **−0.499** ***p*** = **0.002**	*r* = −0.080 *p* = 0.624	***r*** = **−0.461** ***p*** = **0.003**
PD‐L1 TPS	*r* = −0.055 *p* = 0.735	***r*** = **0.672** ***p *< 0.001**		***r*** = **0.406** ***p*** = **0.009**	***r* = 0.336** ***p*** = **0.034**	*r* = −0.234 *p* = 0.175	*r* = 0.093 *p* = 0.569	*r* = −0.280 *p* = 0.082
B7‐1 positive rate	*r* = 0.066 *p* = 0.686	***r*** = **0.461** ***p*** = **0.003**	***r*** = **0.406** ***p*** = **0.009**		*r* = 0.061 *p* = 0.708	*r* = 0.036 *p* = 0.838	*r* = 0.155 *p* = 0.340	*r* = −0.119 *p* = 0.465
PD‐1 positive rate	*r* = −0.042 *p* = 0.799	*r* = 0.104 *p* = 0.524	***r*** = **0.336** ***p*** = **0.034**	*r* = 0.061 *p* = 0.708		*r* = 0.247 *p* = 0.152	*r* = 0.265 *p* = 0.100	*r* = 0.243 *p* = 0.130
CD3 positive rate	*r* = 0.071 *p* = 0.624	***r*** = **−0.499** ***p*** = **0.002**	*r* = −0.234 *p* = 0.175	*r* = 0.036 *p* = 0.838	*r* = 0.247 *p* = 0.152		*r* = 0.238 *p* = 0.168	***r*** = **0.612** ***p *< 0.001**
CD8 positive rate	*r* = −0.008 *p* = 0.962	*r* = −0.080 *p* = 0.624	*r* = 0.093 *p* = 0.569	*r* = 0.155 *p* = 0.340	*r* = 0.265 *p* = 0.100	*r* = 0.238 *p* = 0.168		***r*** = **0.436** ***p*** = **0.005**
CD4 positive rate	*r* = −0.055 *p* = 0.735	***r*** = **−0.461** ***p*** = **0.003**	*r* = −0.280 *p* = 0.082	*r* = −0.119 *p* = 0.465	*r* = 0.243 *p* = 0.130	***r*** = **0.612** ***p *< 0.001**	***r*** = **0.436** ***p*** = **0.005**	

Bold is *p* <0.05.

**TABLE 6:** Correlation between immunohistochemical positive rate, Brinkman index, and age of the patients (Lymph node metastasis)

	Age	Smoking amount	PD‐L1	B7‐1	PD‐1	CD3	CD8	CD4
Age		*r* = 0.116 *p* = 0.477	*r* = −0.118 *p* = 0.475	*r* = 0.198 *p* = 0.227	*r* = 0.099 *p* = 0.565	*r* = 0.175 *p* = 0.308	*r* = −0.278 *p* = 0.867	*r* = 0.272 *p* = 0.095
Smoking amount (Brinkman index)	*r* = 0.116 *p* = 0.477		***r*** = **0.392** ***p *< 0.014**	*r* = 0.309 *p* = 0.055	*r* = −0.093 *p* = 0.589	*r* = 0.182 *p* = 0.287	*r* = 0.105 *p* = 0.553	*r* = −0.190 *p* = 0.245
PD‐L1 TPS	*r* = −0.118 *p* = 0.475	***r*** = **0.392** ***p *< 0.014**		*r* = −0.130 *p* = 0.431	*r* = −0.114 *p* = 0.510	*r* = 0.057 *p* = 0.742	*r* = 0.185 *p* = 0.259	*r* = −0.100 *p* = 0.543
B7‐1 positive rate	*r* = 0.198 *p* = 0.227	*r* = 0.309 *p* = 0.055	*r* = −0.130 *p* = 0.431		*r* = 0.150 *p* = 0.381	*r* = 0.258 *p* = 0.128	*r* = 0.214 *p* = 0.190	*r* = −0.186 *p* = 0.256
PD‐1 positive rate	*r* = 0.099 *p* = 0.565	*r* = −0.093 *p* = 0.589	*r* = −0.114 *p* = 0.510	*r* = 0.150 *p* = 0.381		*r* = 0.249 *p* = 0.142	*r* = 0.010 *p* = 0.955	*r* = −0.020 *p* = 0.907
CD3 positive rate	*r* = 0.175 *p* = 0.308	*r* = 0.182 *p* = 0.287	*r* = 0.057 *p* = 0.742	*r* = 0.258 *p* = 0.128	*r* = 0.249 *p* = 0.142		***r*** = **0.389** ***p*** = **0.019**	*r* = 0.243 *p* = 0.153
CD8 positive rate	*r* = −0.278 *p* = 0.867	*r* = 0.105 *p* = 0.553	*r* = 0.185 *p* = 0.259	*r* = 0.214 *p* = 0.190	*r* = 0.010 *p* = 0.955	***r*** = **0.389** ***p*** = **0.019**		*r* = 0.304 *p* = 0.060
CD4 positive rate	*r* = 0.272 *p* = 0.095	*r* = −0.190 *p* = 0.245	*r* = −0.100 *p* = 0.543	*r* = −0.186 *p* = 0.256	*r* = −0.020 *p* = 0.907	*r* = 0.243 *p* = 0.153	*r* = 0.304 *p* = 0.060	

Bold is *p* <0.05.

### 3.5 B7‐1, PD‐1, CD3, CD4, and CD8 positive rates in primary cancer and lymph node metastases

**FIGURE 3:**
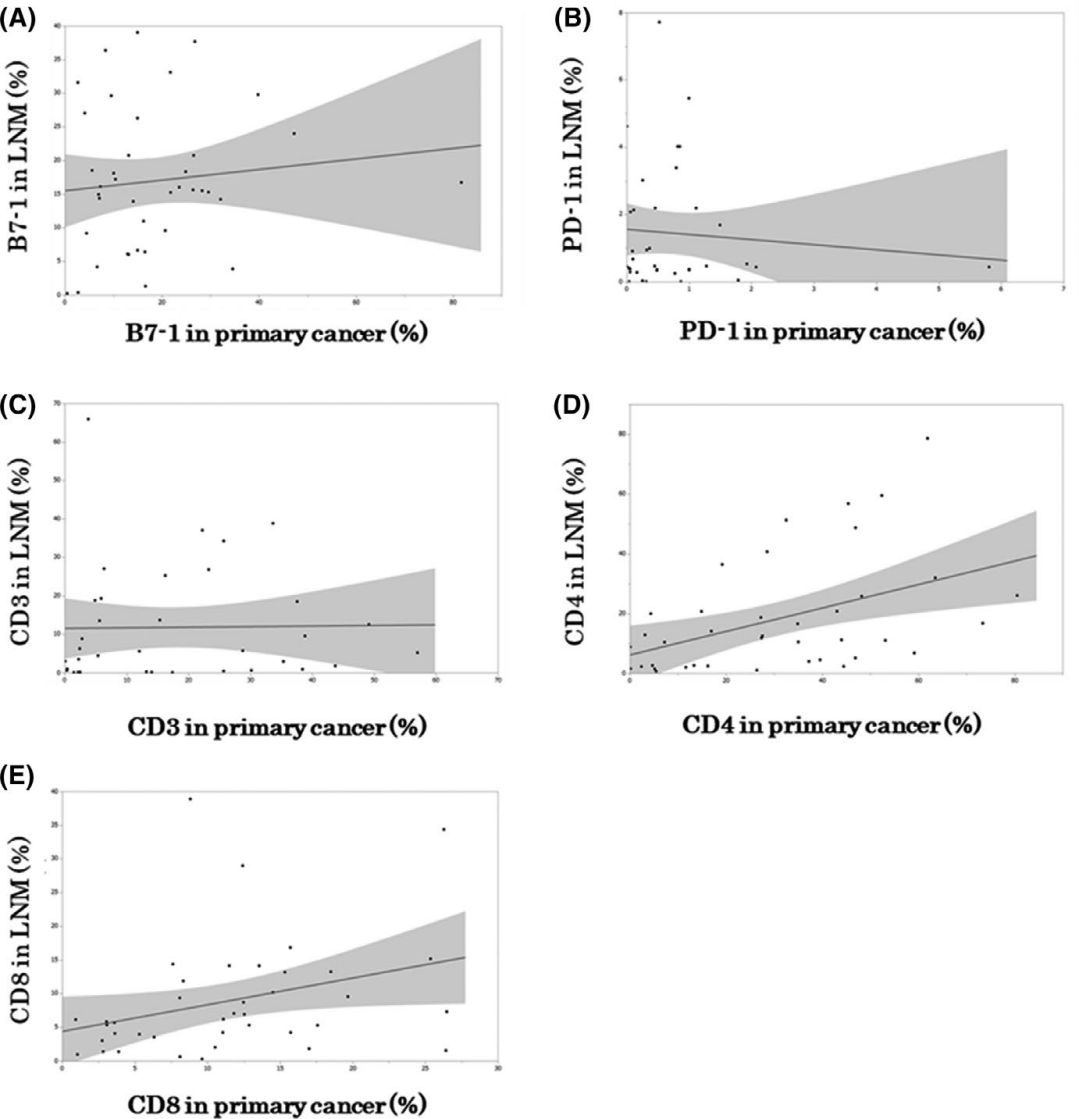
The correlation between rates of target molecules in primary carcinoma and lymph node metastasis lesions. (A) The horizontal axis represents the B7‐1 positive rate of primary lesions (%), and the vertical axis, the B7‐1 positive rate of lymph node metastases. *r *= 0.113 *p* = 0.492, and no significant correlations are detected between the B7‐1 positive rate of the primary carcinoma and lymph node metastatic lesions. (B) The horizontal axis represents the PD‐1 positive rate of primary lesions (%), and the vertical axis, the B7‐1 positive rate of lymph node metastases. *r *= −0.087 *p *= 0.615, and no significant correlations are detected between the B7‐1 positive rate of the primary carcinoma and lymph node metastatic lesions. (C) The horizontal axis represents the CD3 positive rate of primary lesions (%), and the vertical axis, the B7‐1 positive rate of lymph node metastases. *r *= −0.017 *p *= 0.924, and no significant correlations are detected between the CD3 positive rate of the primary carcinoma and lymph node metastatic lesions. (D) The horizontal axis represents the CD4 positive rate of primary lesions (%), and the vertical axis, the CD4 positive rate of lymph node metastases. *r *= 0.448 *p* = 0.004; a significant positive correlation is detected between the CD4 positive rate of the primary carcinoma and lymph node metastatic lesions. (E) The horizontal axis represents the CD8 positive rate of primary lesions (%), and the vertical axis, the CD4 positive rate of lymph node metastases. *r *= 0.324 *p* = 0.044; a significant positive correlation is detected between the CD8 positive rate of the primary carcinoma and lymph node metastatic lesions

We then evaluated the correlation of the positive rates of the molecules examined between primary and metastatic lesions (Figure 3A: B7‐1, B: PD‐1, C: CD3, D: CD4, E: CD8). B7‐1, PD‐1, and CD3 positive rate in lymph node metastasis were not significantly correlated with the positive rate in primary lesions (B7‐1, *p *= 0.492; PD‐1, *p *= 0.615; CD3, *p *= 0.924). CD4 and CD8 positive rate in lymph node metastasis were significantly correlated with that in primary lesions (CD4, *p *= 0.004; CD8, *p* = 0.044).

### 3.6 PD‐L1 TPS in primary and lymph node metastases

**FIGURE 4:**
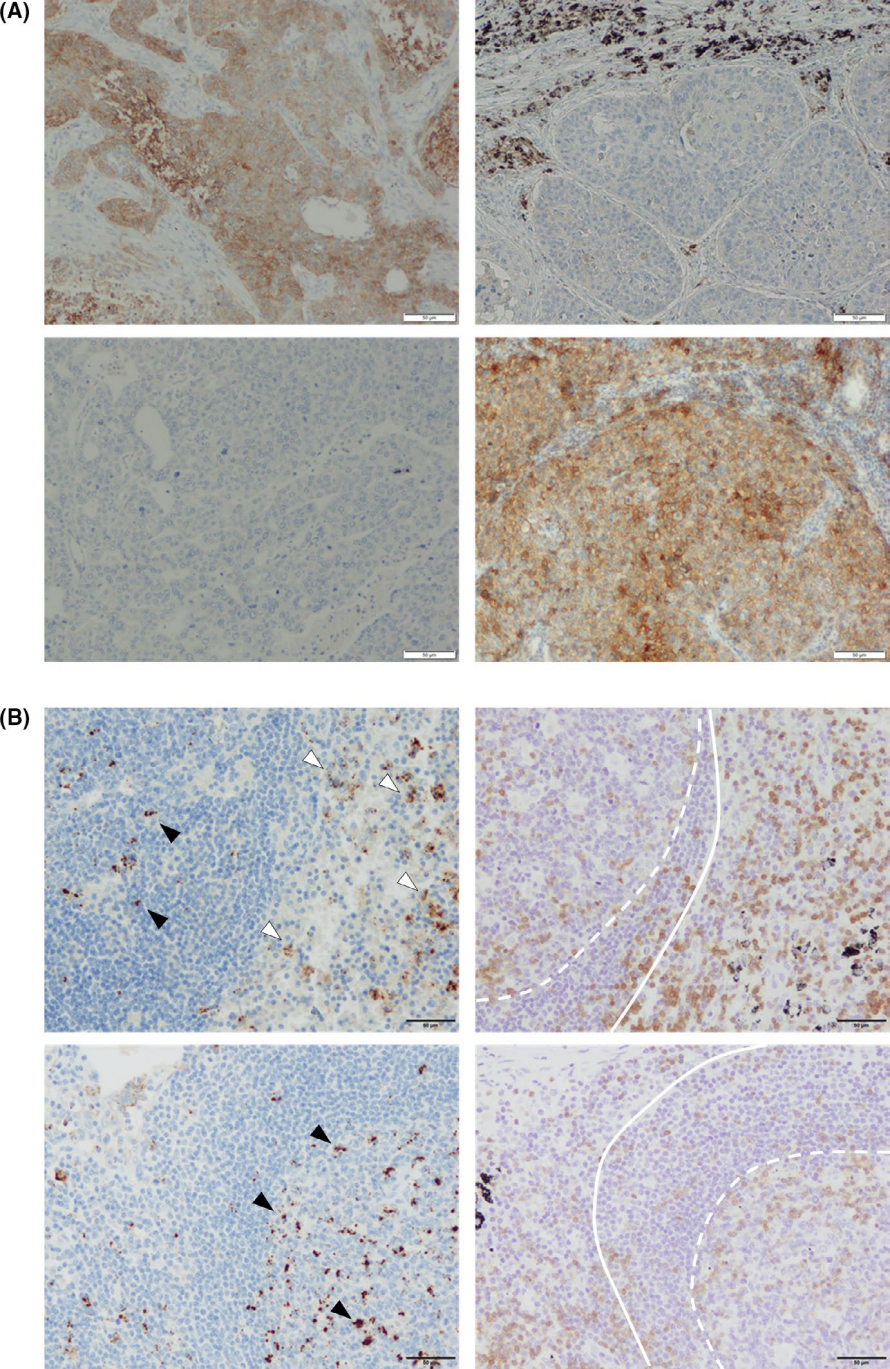
Immunohistochemistry of PD‐L1 in primary site, lymph node metastatic site, and non‐cancer metastatic lymph node. (A) PD‐L1 immunohistochemistry in primary site (*left*) and lymph node metastatic site (*right*) of lung adenocarcinoma case. Upper case is PD‐L1 positive in primary site but not in lymph node metastatic site. The cases in which PD‐L1 is positive only in the primary site (*upper*) and only in the lymph node metastatic site (*lower*) are presented, respectively. The brownish cells are PD‐L1‐positive lung cancer cells (upper left and lower right). The black area in the upper 1/3 of the upper right image is the carbon powder deposition in the non‐cancerous area. (B) Immunohistochemistry of PD‐L1 (*left*) and CD3 (*right*) in pulmonary lymph node without cancer metastasis of lung adenocarcinoma cases. The inside of the solid line is the lymphoid follicle and the inside of the dashed line is the germinal center. The area outside the solid line is the interfollicular region or paracortex. Black arrowheads, PD‐L1‐positive cells in the germinal center; white arrowheads, PD‐L1‐positive cells in the paracortex

There were no significant differences in PD‐L1 TPS between primary and lymph node metastatic lesions in 18 cases but the difference of more than 10% was also detected in 22 cases. In particular, the difference of more than 40% was detected in seven cases (Table 7, Figure 4A). The patients were tentatively classified into two groups according to the status of PD‐L1 TPS, that is, increased and decreased/unchanged TPS in lymph node metastasis compared to primary cancer lesions. B7‐1, PD‐1, CD3, CD4, and CD8 positive rates in primary tumors in the two groups were subsequently compared (Table 8). No significant differences in B7‐1 and PD‐1 positive rates were detected between these two groups. CD8 positive rate was significant higher in the group in which PD‐L1 TPS increased in lymph node metastases compared to decreased/unchanged group (*p *= 0.037). We also further explored the immunolocalization of PDL‐1 in lymph nodes of 10 cases without carcinoma metastasis, in which the germinal centers were histologically discernible without any difficulties (Figure 4B). PD‐L1‐positive cells were predominantly detected in the area where CD3‐positive cells were present.

**TABLE 7:** Difference in PD‐L1 TPS between primary carcinoma and lymph node metastatic lesions

PD‐L1 TPS	Difference between primary cancers and metastases (%)	Number of cases (%)
Metastases > primary cancers	40 ≦	3 (7.5)
	10–30	6 (15.0)
Metastases = primary cancers	0	18 (45.0)
Metastases < primary cancers	10–30	9 (22.5)
	40 ≦	4 (10.0)

Bold is *p* <0.05.

**TABLE 8:** B7‐1, PD‐1, CD3, CD4, and CD8 positive rates of primary tumors in two groups: a group in which PD‐L1 TPS higher in lymph node metastases than primary cancers, and those not

	PD‐L1 TPS (Mean ± SD)		
	Lymph node metastasis > primary cancer (%)	Lymph node metastasis ≦ primary cancer (%)	*p* value
B7‐1 positive rate	14.11 ± 9.21	19.39 ± 16.02	0.487
PD‐1 positive rate	1.22 ± 2.05	0.60 ± 0.59	0.714
CD3 positive rate	23.57 ± 17.32	16.39 ± 15.81	0.284
CD4 positive rate	39.42 ± 23.36	28.90 ± 21.36	0.289
CD8 positive rate	15.89 ± 5.39	10.58 ± 7.21	**0.037**

## 4 DISCUSSION

In this study, we first demonstrated the interaction of PBMC with lung adenocarcinoma cells via soluble factors using a co‐culture system.^14^ The co‐culture study reveal the changes in the expression status of the genes compared to the PBMC monoculture. The genes that increased with fivefold following co‐culture with all lung adenocarcinoma cell lines were *CD80* (B7‐1), *IL*‐*7*, *IL*‐*15*, and *TNF*. Among those, B7‐1 is known to activate T‐cell activation by binding to CD28 and suppress T cells by binding to CTLA‐4, which could subsequently modulate local immunity.^12^ IL‐7 and IL‐15 were both cytokines that work on differentiation, proliferation, and survival of cytotoxic T cells.^22^, ^23^ TNF is also well known to be involved in the production of inflammatory mediators and antibody production in plasma cells.^24^ All of those above could induce tumor immunity and immunity against adenocarcinoma cells was considered to be induced in this model system of co‐culture. However, both IL‐7 and IL‐15 act on the induction of PD‐1 and PD‐L1,^9^ and TNF, especially TNF‐α, was also reported to enhance the expression of PD‐L1.^25^ On the other hand, B7‐1 acts as an immune checkpoint molecule similar to PD‐1 by binding to PD‐L1.^11^ Therefore, results of our present study first reveal that the interaction between carcinoma and immune cells could alter the expression of immune checkpoint‐related molecules in both carcinoma and immune cells, such as PD‐L1, PD‐1, and B7‐1. Exact mechanisms of this alteration above could not be elucidated by results of our present study alone but humoral or soluble factors derived from carcinoma cells themselves were reasonably postulated to induce the immune response, although further investigations are required for clarification.

We then immunolocalized B7‐1 in human NSCLC tissues. B7‐1 was more highly detected in squamous cell carcinoma than in adenocarcinoma, and significantly positively correlated with Brinkman index of the patients and PD‐L1 status in tumor cells. The status of PD‐L1 was reported to be also positively correlated with Brinkman index, and more abundant in squamous cell carcinoma than in adenocarcinoma.^26^ Smoking is also well known to induce numerous gene mutations,^27^ subsequently resulting in T‐cell response to those cells harboring gene mutations.^28^ Subsequently, cytokine secreted from T cells in response to those tumor cells was increased, and those cytokines including IL‐2, IL‐7, IL‐15, and interferon gamma (IFN‐g), could induce PD‐L1 expression.^29^ IFNy has been reported to induce B7‐1 in addition to PD‐L1.^30^ Results of our present study indicate that the same cytokines, for example, IFN‐g, could induce both PD‐L1 in carcinoma cells and B7‐1 in immune cells. In addition, lung squamous cell carcinoma is a common histological type in smokers.^31^ This could account for the correlation detected among B7‐1 expression, PD‐L1 TPS, smoking amount, and histological type in our present study,

In our present study, B7‐1 status and PD‐1 status were not necessarily correlated between primary and lymph node metastatic lesions. On the other hand, the characteristics such as the number of T lymphocytes and the cell types in the tumor stroma of the primary tumor lesions were histopathologically almost the same as in metastatic lesions but of particular interest, the status of immune checkpoint molecules in immune cells was altered from primary lesions in lymph node metastases. These results suggest that the status of immune checkpoint molecules in lymphocytes could be changed by humoral or soluble factors derived from carcinoma cells in the lymph node metastases.

In our present study, some NSCLC cases had different PD‐L1 TPS between metastatic and primary lesions. This difference in PD‐L1 TPS between primary cancer and lymph node metastasis was reported to be approximately 10%–20%.^4^, ^5^ In these studies, differences in PD‐L1 TPS were attributed to intratumoral heterogeneity of PD‐L1 without any references to the difference in local tissue microenvironment. In our present study, NSCLC cases harboring relatively abundant PD‐L1 TPS in metastatic regions compared to primary cancer regions turned out to harbor much higher CD8‐positive cells infiltration in primary tumor. Therefore, PD‐L1 TPS is considered to be much higher in metastases in the cases harboring abundant infiltration of CTL in tissue microenvironment of primary lesion. The study using a mouse tumor model also demonstrated that exposure to cytotoxic T cells with IFN‐g induce the development of immune resistance through genetic evolution of tumor cells.^32^ These results as well as those of our present study indicate that patients with relatively abundant CTLs infiltrating in the tumor could have the potential to induce PD‐L1 in carcinoma cells even if the primary tumor had relatively low PD‐L1 status in carcinoma cells. As described above, the status of CTLs infiltrating into the tumor was by no means different between primary and metastatic lesions of NSCLC patients. These results also suggested that the tumors could acquire PD‐L1 expression as a result of alterations of the surrounding immune microenvironment in lymph node metastases. In addition, results from Study 1 in our present study demonstrate that the gene expression of PD‐L1‐induced cytokines derived from lymphocytes was indeed increased under the presence of carcinoma cells. Therefore, the interaction between carcinoma cells and lymphocytes, especially CTL, in metastatic lesions is reasonably postulated to be involved in the changes in the expression profiles of PD‐L1. Changes in the status of PD‐L1 in metastatic carcinomas could therefore contribute to the therapeutic efficacy of PD‐1/PD‐L1 inhibitors in some NSCLC patients. Therefore, the analysis of CTLs as well as B7‐1 status in primary tumor tissues could provide further information as to the necessity of evaluating PD‐L1 status in metastasis or liquid biopsy when applicable in order to improve the therapeutic efficacy of PD‐1/PD‐L1 inhibitors in NSCLC patients.

In lymph nodes of NSCLC patients without metastases, PD‐L1‐positive cells were detected in the areas where CD3‐positive cells were present. There are by no means any carcinoma cells in those regions of those lymph nodes but priming by antigen‐presenting cells is considered to occur here according to the circulating cancer antigens. In the OAK study, the patients in the PD‐L1 (evaluated by SP‐142 antibody) low or undetectable subgroup (TC0IC0) were also reported to have improved survival with atezolizumab.^33^ Therefore, the action point of PD‐L1 inhibition could also be involved in suppression of priming in lymph nodes without any carcinoma cells present as well as PD‐L1 acquisition of carcinoma cells in lymph node metastatic lesions described above. The tumor microenvironment could also be dramatically altered in distant metastases. The dynamics of immune checkpoint‐related factor expression in the distant metastatic sites of lung cancer have remained unclear. In this study, we focused on lymph node metastases, but further investigation including the exploration of distant metastatic sites is required for the development of lung cancer immunotherapy.

## ETHICAL APPROVAL STATEMENT

The study protocol was approved by Tohoku University School of Medicine Ethics Committee (2020‐1‐176).

## Data Availability

Data available upon request due to privacy/ethical restrictions.
